# Professional Ethics

**Published:** 1912-06

**Authors:** 


### Professional Ethics

THE confession of deliberate murder of a supernumerary fiancee by the Rev. Mr. Richeson, the implication of an entire family in the disgrace, without blame on their part, the hint of other social tragedies stopping short of murder, the final step by which the community rids itself of a dangerous member, make a sad story, on whose obvious morals, we need not dwell.

A medical journal may, however, be pardoned for calling attention to the wisdom of maintaining, not merely ideals of personal and professional ethics, but a definite system of codification of ethical principles, of censorship of professional conduct and of applying this system in such a way as to prevent, if possible, extreme results of carelessness, temptation and wrong individual proclivities. We can all call to mind physicians of good standing and of useful and honorable careers, who have been kept to professional standards and who have been saved from permanent lapses, by the prompt, perhaps severe, action of this system. Essentially the same system is carried out in the great churches, while in many other denominations, the individual clergyman is left almost wholly to his own guidance until a catastrophy has occurred. In these days when the influence of than many upright clergymen can counteract by whole lives of precept and good example. In the law and other professions we find, of course, punitive methods but no such elaborate, constantly guiding system of ethics as that for whose maintenance the medical profession is so often ridiculed.

